# TCR β CDR3 repertoire remodeling in pediatric myocarditis reveals clonal expansion and disease-associated public clonotypes

**DOI:** 10.3389/fimmu.2026.1711681

**Published:** 2026-02-04

**Authors:** Xixiong Lin, Xing Zhang, Liping Zhang, Linhu Hui, Zhongjian Su, Xingzhu Liu, Bin Li, Jun Li, Yanfei Chen

**Affiliations:** 1 Department of Cardiology, Kunming Children’s Hospital, Kunming, Yunnan, China; 2 Department of Immunology, Center of Immunomolecular Engineering, Zunyi Medical University, Zunyi, China

**Keywords:** antigen specificity, CDR3 diversity, clonal expansion, pediatric myocarditis, TCR repertoire

## Abstract

**Background:**

Pediatric myocarditis is an inflammatory disease of the heart with heterogeneous clinical presentations and poorly understood immune mechanisms. T cell receptor (TCR) repertoire profiling provides insights into disease-associated adaptive immune responses.

**Methods:**

We performed high-throughput sequencing of TCR β chain CDR3 repertoires from 28 peripheral blood samples of pediatric myocarditis patients (Myo) and nine age-matched healthy controls (NC). Clonal diversity, V and J gene usage, CDR3 length distribution, clonotype sharing, and antigen-specific annotations were systematically analyzed.

**Results:**

The Myo group exhibited significantly reduced clonal diversity as measured by D50 and Chao1 indices, accompanied by expansion of large clones and reduced representation of small clones. Distinct biases in V and J gene usage were observed, with increased TRBV14, TRBV28, TRBJ1-1, TRBJ1-2, TRBJ1-5, TRBJ1-6, and TRBJ2-2, and decreased TRBV9, TRBJ2-4, TRBJ2-5, and TRBJ2-7. CDR3 length distribution showed an enrichment of longer sequences in myocarditis patients, alongside altered nucleotide insertions/deletions and amino acid usage. Clonotype sharing was markedly higher in the Myo group, and 16,460 public clonotypes were detected in ≥10 patients. Database annotation revealed an enrichment of matches to pathogen-associated TCR records, predominantly associated to Mycobacterium tuberculosis, influenza, cytomegalovirus, and Epstein–Barr virus. Seventeen high-frequency clonotypes were highlighted as candidate myocarditis-related TCR signatures based on database matches.

**Conclusions:**

Our study demonstrates distinct repertoire remodeling in pediatric myocarditis, characterized by reduced diversity, skewed V/J gene usage, biased CDR3 composition, and enriched public clonotypes. These findings provide novel insights into disease-related adaptive immune responses and may inform biomarker discovery for diagnosis and therapeutic strategies.

## Introduction

Myocarditis in children represents a significant clinical challenge, often presenting with non-specific symptoms but carrying the risk of rapid progression to fulminant myocarditis with cardiogenic shock or malignant arrhythmias (1, 2). Early diagnosis is difficult, current therapeutic options are limited, and long-term prognosis remains unpredictable (3, 4). These challenges highlight the urgent need to better understand the immunopathological mechanisms of pediatric myocarditis (5, 6), which may open new avenues for early detection and targeted interventions.

Increasing evidence indicates that immune dysregulation, rather than direct viral cytotoxicity alone, plays a decisive role in myocardial injury. Infiltration of CD4^+^ and CD8^+^ T lymphocytes and macrophages is a hallmark of lymphocytic myocarditis (7). While CD8^+^ T cells contribute to viral clearance, their excessive activation promotes myocardial damage, as demonstrated in both experimental models and clinical cases (8). In fulminant myocarditis, CD8^+^ T cells exhibit pronounced clonal expansion, heightened cytotoxicity, and increased chemotaxis, with CXCL12/CXCR4 signaling driving their hyperactivation. Pharmacologic blockade of CXCR4 mitigates T cell accumulation and alleviates cardiac injury in mice (8). Similarly, immune checkpoint inhibitor–associated myocarditis is characterized by expansion of effector CD8^+^ T cells, underscoring the central role of T cell–mediated immune responses (9).

The T cell receptor (TCR) repertoire provides a molecular record of antigen exposure and immune selection (10). Diversity is primarily shaped by the complementarity-determining region 3 (CDR3) of the β chain, which determines antigen specificity. Antigen-driven clonal expansion and skewed V gene usage have been reported in myocarditis, supporting the involvement of superantigen-driven or pathogen-specific responses (11–13). Single-cell transcriptomic and repertoire analyses further reveal enhanced cytotoxic programs and clonal dominance in both viral and autoimmune myocarditis (13, 14).

Despite these insights, the TCR CDR3 repertoire in pediatric myocarditis remains poorly defined. Given that children have an immature immune system, their T cell responses may differ fundamentally from adults (15, 16). High-throughput sequencing (HTS) of TCR CDR3 regions enables comprehensive characterization of clonal architecture and identification of disease-associated public TCRs (17). Compared with conventional methods such as flow cytometry or spectratyping, HTS provides substantially higher sensitivity and resolution, allowing precise detection of clonal expansion, repertoire diversity, and low-frequency clonotypes at the sequence level. Here, we systematically profiled the TCR CDR3 repertoire in pediatric myocarditis, aiming to delineate clonal expansion, diversity, and shared sequences, thereby advancing mechanistic understanding and facilitating future studies toward potential diagnostic and therapeutic applications.

## Materials and methods

### Study population

The pediatric patients diagnosed with myocarditis (Myo) at Kunming Children’s Hospital between July 2022 and May 2023 were collected. The inclusion criteria were based on the Recommendations for the Diagnosis of Pediatric Myocarditis (2018 edition), specifically the diagnostic criteria for acute viral myocarditis. Exclusion criteria included myocardial injury caused by drugs, toxins, or systemic autoimmune diseases; hypertensive heart disease, primary cardiomyopathy, valvular heart disease, and other organic heart diseases; coexisting β-receptor hyperfunction or hyperthyroidism; and disease duration >30 days from onset to the appearance of cardiac symptoms.

A total of 28 pediatric myocarditis cases were initially recruited, of which 17 samples passed library preparation and sequencing quality control and were included in the final analysis. In addition, nine age-matched healthy children without myocarditis were enrolled as controls. The median age of the included patients was 6.5 years (range 1.08–14.75), with 8 males and 9 females; no participants had chronic comorbidities. All patients were sampled during the acute phase and prior to any antiviral, glucocorticoid, or immunosuppressive treatment to avoid treatment-related immune alterations. Peripheral blood samples were obtained within 24 hours of hospital admission according to the standardized institutional clinical and laboratory protocol of Kunming Children’s Hospital. Peripheral blood mononuclear cells (PBMCs) were isolated using Ficoll-Paque density gradient centrifugation. This study was approved by the Ethics Committee of Kunming Children’s Hospital, and written informed consent was obtained from the parents or legal guardians of all participants.

### Sample collection

Peripheral blood (2 mL) was collected into EDTA-anticoagulated vacutainer tubes. Samples were used to isolate peripheral blood mononuclear cells (PBMCs) by Ficoll-Paque density gradient centrifugation, followed by red blood cell lysis. PBMCs were resuspended in serum-free cryopreservation medium and stored in liquid nitrogen until further processing.

### RNA extraction and cDNA synthesis

PBMCs were thawed at 37 °C, and total RNA was extracted using TRIzol™ Reagent (Invitrogen, Cat. No. 15596026) following the manufacturer’s instructions. The concentration and purity of RNA were assessed with a NanoDrop 2000 spectrophotometer (Thermo Fisher Scientific), and RNA integrity was evaluated by agarose gel electrophoresis. Complementary DNA (cDNA) was synthesized from 1 µg of total RNA using the PrimeScript™ RT Reagent Kit with gDNA Eraser (Takara, Cat. No. RR047A) according to the manufacturer’s protocol. The resulting cDNA was stored at –20 °C until use.

### PCR amplification of TCR fragments and High-throughput sequencing

cDNA was subjected to multiplex PCR using forward primers targeting the TCR Vβ region and reverse primers targeting the Cβ region. Each reaction mixture contained cDNA template, DreamTaq™ Green PCR Master Mix (2×, Thermo Fisher Scientific, Cat. No. K1081), primers (**Supplementary Table S1**), and nuclease-free water. PCR products were verified by 2% agarose gel electrophoresis with ethidium bromide staining, and the expected bands were excised and purified using the QIAquick Gel Extraction Kit (Qiagen, Cat. No. 28704). The concentration and purity of purified amplicons were measured with a NanoDrop 2000 spectrophotometer (Thermo Fisher Scientific), and samples were sequenced on the DNBSEQ platform (BGI Genomics, Shenzhen, China) to generate high-throughput TCR β CDR3 repertoire data. All samples were processed using the same library preparation protocol and the same sequencing protocol to minimize technical batch effects.

### Bioinformatics analysis

Raw sequencing data in FASTQ format were first subjected to quality control using fastp (v0.23.2) for adapter trimming, removal of low-quality reads (Q<20), and filtering of short reads (<50 bp). Clean reads were processed using MiXCR (v3.0.13) (18) with the analyze amplicon pipeline and default parameters to perform V(D)J alignment against the IMGT reference database (19), clonotype assembly, and identification of productive (functional) TCRβ CDR3 sequences. MiXCR was configured to output productive sequences only.

To ensure the accuracy of clonotype assignment, a secondary filtering step was performed after MiXCR processing. Entries containing non-standard placeholder symbols (e.g., “_”) or CDR3 sequences not starting with cysteine (C) or not ending with phenylalanine (F) were removed. Only high-confidence, in-frame productive CDR3 sequences without stop codons were retained as the final “usable sequence set” for downstream analysis.

In this study, a clonotype was defined as sequences sharing the same V gene, the same J gene, and a CDR3 amino acid sequence with ≤2 amino acid mismatches. This definition accounts for both V/J gene usage and minor variations potentially arising from sequencing noise or somatic mutations, enabling biologically meaningful aggregation of closely related T-cell clones.

Subsequent repertoire analyses were performed primarily using the Immunarch R package (v0.6.8) (https://immunarch.com/index.html), unless otherwise specified. Diversity indices (Shannon, Simpson, Chao1) and clonality were calculated using Immunarch with default settings. V, D, and J gene usage, CDR3 length distribution, and repertoire overlap across samples were also assessed using Immunarch integrated functions. V–J gene pairing analysis was carried out using VDJtools (v1.2.1) (20).

### Nucleotide insertions and deletions at V–D–J junctions

Nucleotide deletion and insertion events at TCR β CDR3 junctions were analyzed for each clonotype. Specifically, V3′D and J5′D indicate the number of nucleotides deleted at the 3′ end of the V gene and 5′ end of the J gene, respectively, while V3′I and J5′I indicate the number of nucleotides inserted at the same positions. These metrics were calculated based on the alignment of each CDR3 sequence to its corresponding germline V, D, and J gene segments using the IMGT reference sequences.

### Functional annotation of CDR3 sequences

Functional annotation of CDR3 sequences was conducted using the McPAS-TCR (21) and VDJdb databases (22). Exact CDR3 amino acid sequence matching (no motif-based expansion) was applied to retrieve previously reported antigen-reactive TCR records. Only human entries with documented experimental evidence were retained, and motif-only or predicted annotations were excluded. V-gene or HLA matching was not used in this analysis.

### Statistical analysis

Statistical analyses were performed using GraphPad Prism. Continuous variables with normal distribution were expressed as mean ± standard deviation (SD), and differences between two groups were compared using independent-sample t test or Welch’s t test. Non-normally distributed variables were presented as median (P25, P75), and compared by Mann–Whitney test. Data visualization, including repertoire landscape plots and statistical graphics, was conducted using GraphPad Prism, while figures were refined and formatted with Adobe Photoshop. A p-value < 0.05 was considered statistically significant.

## Results

### TCR β repertoire sequencing summary

High-throughput sequencing of TCR β CDR3 repertoires generated 3.6 to 13.1 million raw reads per sample, of which a substantial proportion could be successfully aligned to reference germline genes. On average, 30–70% of reads were assembled into productive clonotypes, providing tens of thousands to nearly half a million clonotypes per sample. Among these, unique clonotypes typically accounted for a considerable fraction, indicating adequate repertoire coverage (**Supplementary Table S2**).

### Diversity of the TCR-β CDR3 repertoire

**Figure 1:**
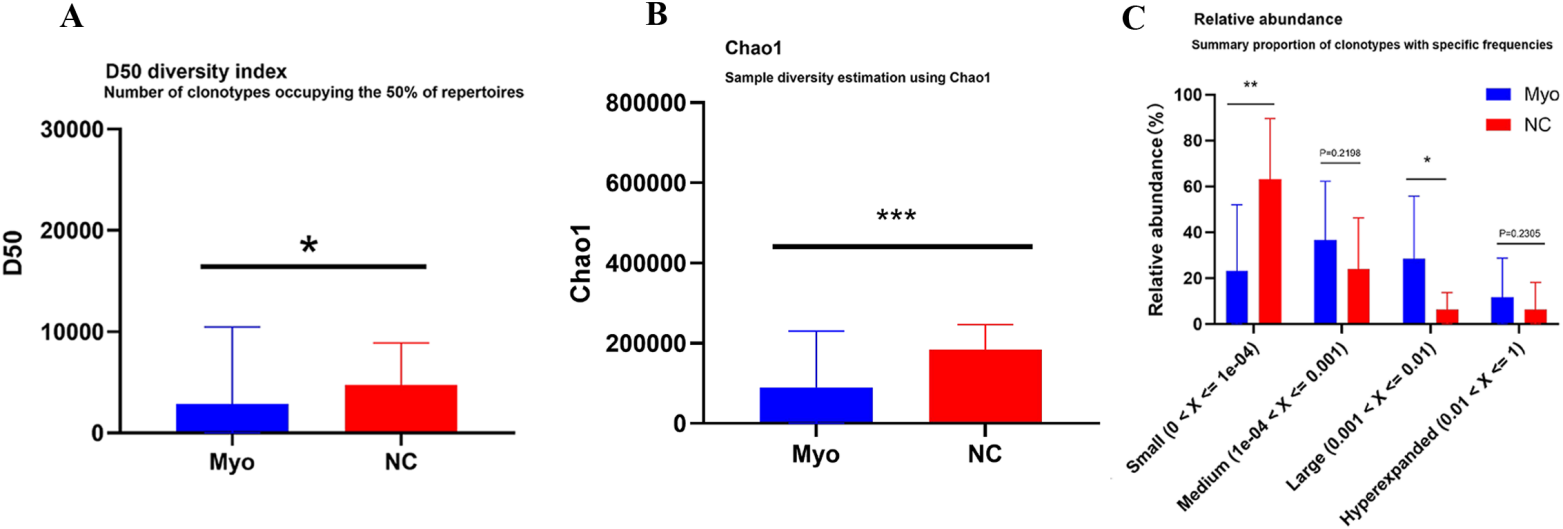
Clonal diversity and clonotype distribution of TCR β CDR3 repertoires. **(A)** D50 index for repertoire diversity analysis between Myo and NC groups; **(B)** Chao1 estimator for repertoire diversity analysis between Myo and NC groups; **(C)** Clonotype abundance distribution among small, medium, large and hyperexpanded clones. Myo, pediatric myocarditis; NC, normal control; *p < 0.05, **p < 0.01, ***p < 0.001.

To assess clonal diversity, we quantified repertoire diversity using the D50 index (**Figure 1A**) and Chao1 estimator (**Figure 1B**), both of which consistently indicated a reduced TCR diversity in the Myo group relative to NC. Additional diversity indices further confirmed this trend and are provided in **Supplementary Figure S1** for completeness. To identify which clones contributed to this reduction, clonotype abundance was analyzed (**Figure 1C**). Expanded large clones were significantly enriched in the Myo group, while the proportion of small clones was markedly decreased. Visualization of the top 200 clonotypes further supported this observation. The Myo group displayed highly expanded dominant clones and less uniform distribution, consistent with skewed clonal expansion.

### V and J gene usage patterns

**Figure 2:**
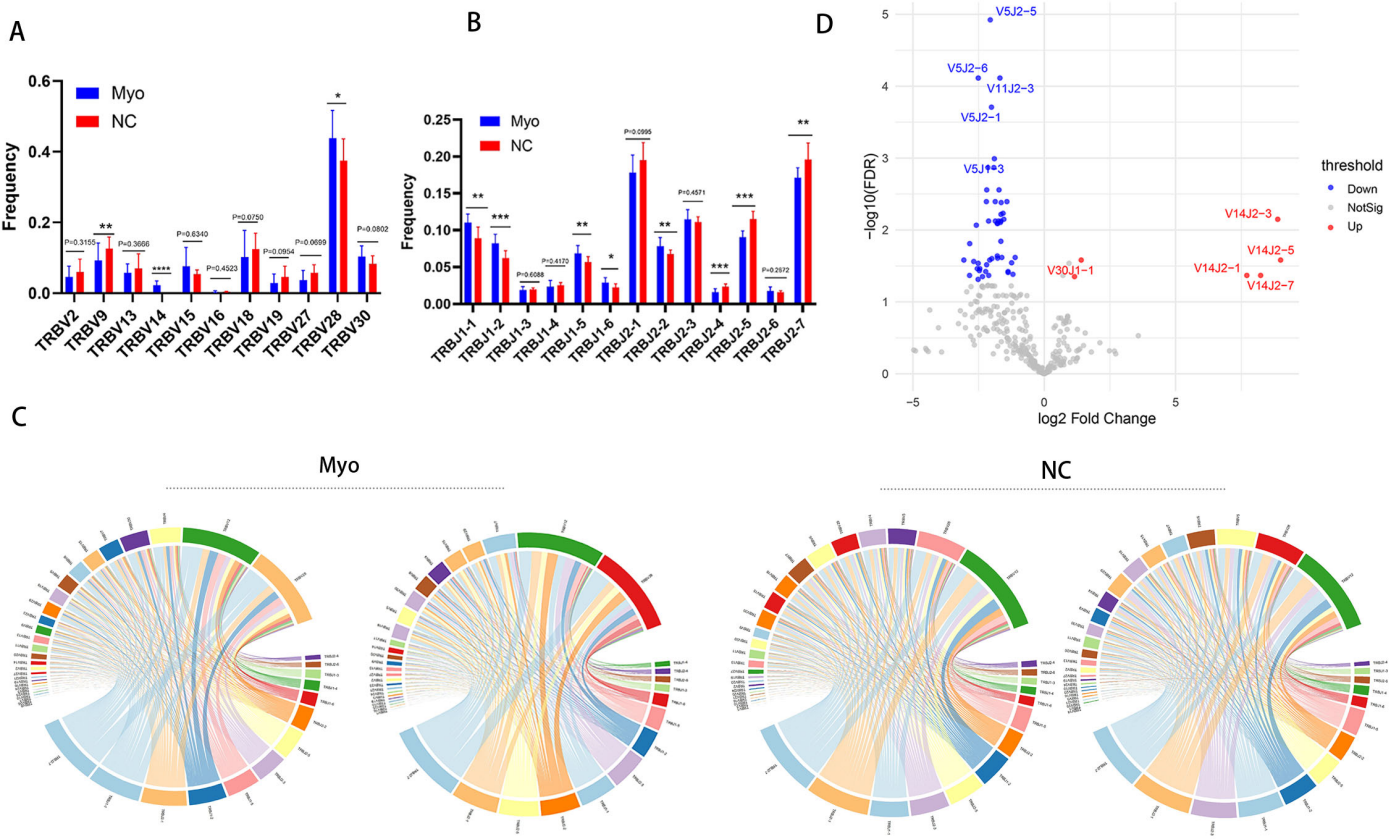
V and J gene usage and V–J pairing patterns of TCR β CDR3 repertoires. **(A)** TRBV gene usage frequencies between Myo and NC groups; **(B)** TRBJ gene usage frequencies between Myo and NC groups; **(C)** Representative circos plots of V–J gene pairing from individual samples; **(D)** Volcano plot of V–J pairing frequencies showing significantly enriched and reduced combinations in the Myo group compared with NC. Myo, pediatric myocarditis; NC, normal control; *p < 0.05, **p < 0.01, ***p < 0.001.

The diversity of V and J gene segment utilization contributes substantially to the overall repertoire heterogeneity. To explore differences between groups, we quantified V and J gene usage frequencies across all samples. Both cohorts preferentially employed TRBV12-3 (**Supplementary Figure S2**). We grouped V genes at the IMGT-defined gene family level and performed statistical comparisons, which revealed significant group-specific biases in V gene family usage. In the Myo group, TRBV14 and TRBV28 were overrepresented, whereas TRBV9 was underrepresented (**Figure 2A**). Similarly, TRBJ1-1, TRBJ1-2, TRBJ1-5, TRBJ1-6, and TRBJ2–2 were increased, while TRBJ2-4, TRBJ2-5, and TRBJ2–7 were decreased (**Figure 2B**). V–J pairing analysis revealed characteristic recombination patterns. Representative circos plots from individual samples (**Figure 2C**) and pooled group-level data (**Figure 2D**) showed preferential expansion of TRBV14-TRBJ2-1, TRBV14-TRBJ2-3, TRBV14-TRBJ2-5, TRBV14-TRBJ2-7, and TRBV30-TRBJ1–1 in Myo patients, whereas combinations such as TRBV5-TRBJ1-3, TRBV5-TRBJ2-1, TRBV5-TRBJ2-5, TRBV5-TRBJ2-6, and TRBV11-TRBJ2–3 were reduced.

### CDR3 length distribution and amino acid usage

**Figure 3:**
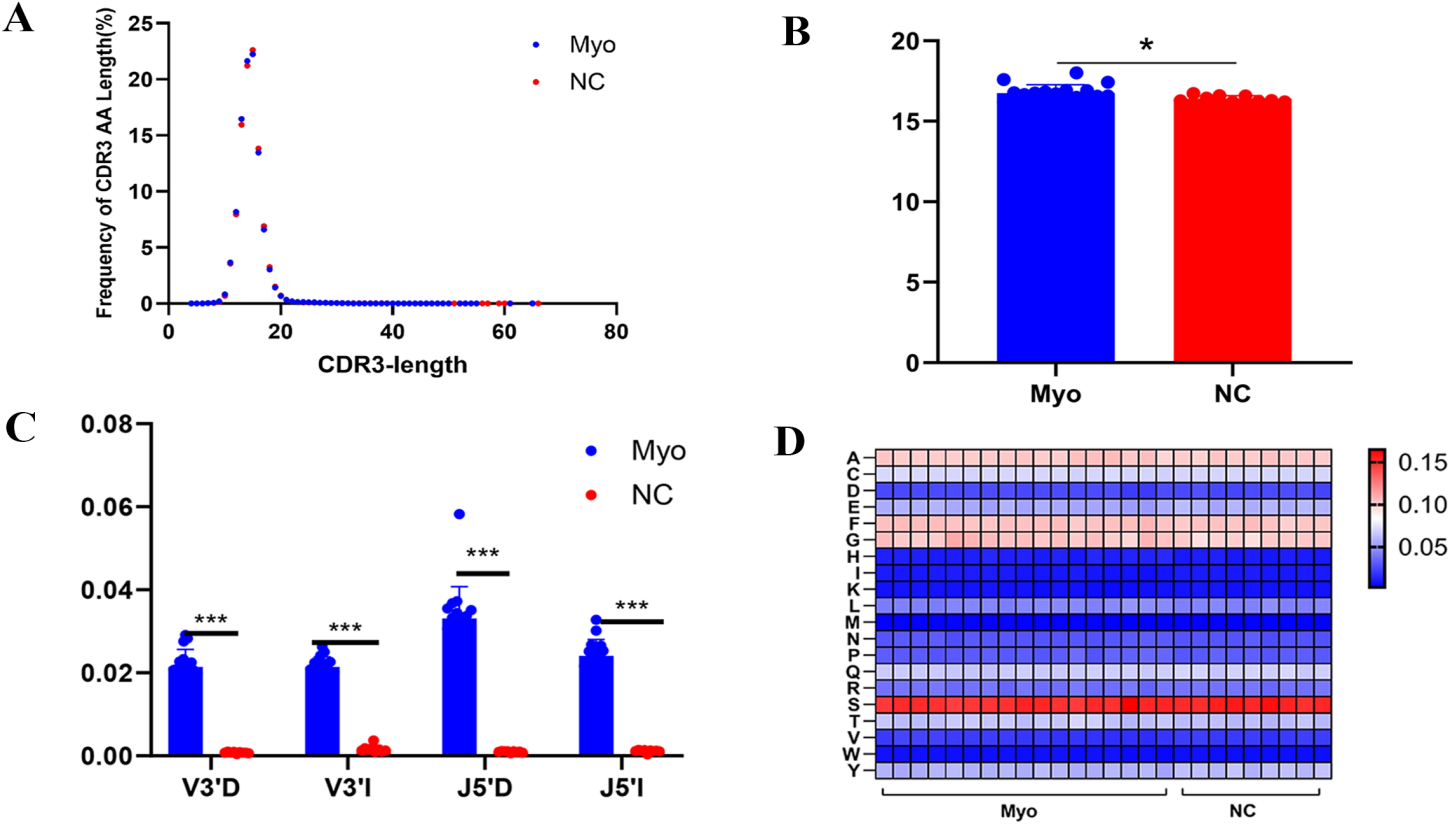
Characteristics of CDR3 length and composition in TCR β CDR3 repertoires. **(A)** Distribution of CDR3 lengths in the Myo and NC groups; **(B)** Proportion of CDR3 longer than the mean length (14.8 amino acids); **(C)** Nucleotide insertion and deletion events at V3’D, V3’I, J5’D, and J5’I sites; **(D)** Heatmap of amino acid usage frequencies in CDR3 regions. V3’D and J5’D represent deletions at the 3’end of the V gene and 5’end of the J gene, respectively; V3’I and J5’I represent insertions at the same positions. Myo, pediatric myocarditis; NC, normal control. *p < 0.05, ***p < 0.001.

The CDR3 region, typically 10–20 amino acids in length, is critical for TCR specificity and clonal heterogeneity. Analysis of CDR3 length distribution (**Supplementary Figure S3**; **Figure 3A**) revealed a Gaussian-like peak at 15 amino acids in both groups. However, the proportion of CDR3s longer than the mean (14.8 AA) was significantly higher in the Myo group (**Figure 3B**). Examination of recombination-associated nucleotide insertions and deletions showed increased events at V3’D, V3’I, J5’D, and J5’I in Myo samples (**Figure 3C**). At the amino acid level, modest but statistically significant shifts in CDR3 composition were observed between groups. In the Myo cohort, the relative frequencies of F, G, H, and T showed an increasing trend, whereas E, K, Q, S, and Y exhibited a decreasing trend compared with NC (**Figure 3D**; **Supplementary Table S3**). Although the magnitude of change was limited and influenced by inter-individual variability, these patterns suggest potential disease-associated biases in CDR3 amino acid usage.

### Shared clonotypes

**Figure 4:**
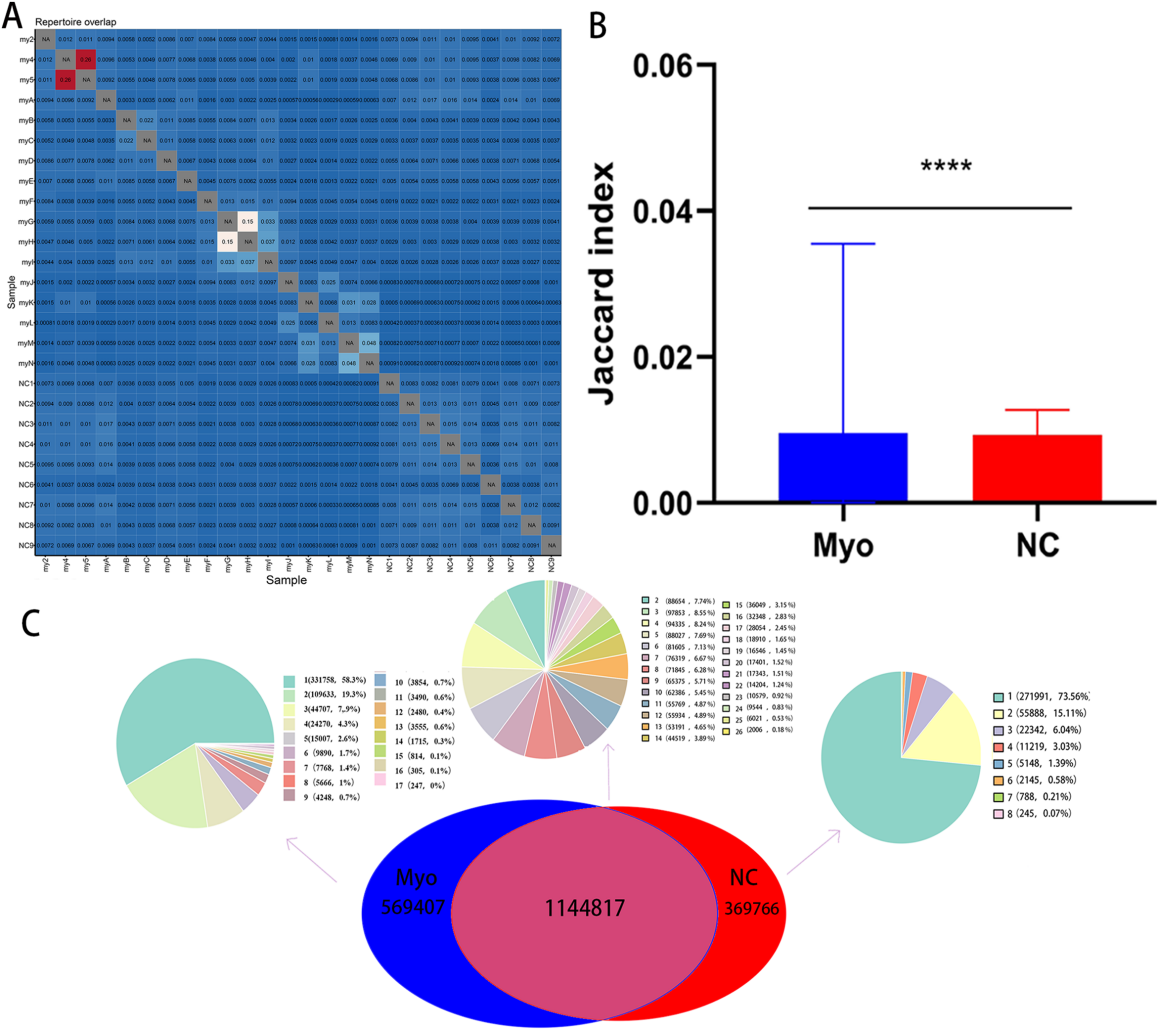
Clonotype sharing across individuals in Myo and NC groups. **(A)** Clonotype overlap matrix among individuals within each group; **(B)** Jaccard index analysis of shared clonotype frequencies between the Myo and NC groups; **(C)** Venn diagram showing the number of common and unique CDR3 sequences across groups. Myo, pediatric myocarditis; NC, normal control. Asterisks indicate statistical significance.

Shared CDR3 sequences across individuals provide insights into disease-related immune responses. As shown in **Figures 4A, B**, clonotype overlap was higher among Myo patients than NC, with overall increased sharing within the disease group. Across groups, 1,144,817 CDR3 sequences were common to both cohorts (**Figure 4C**). In the Myo group, clonotypes shared by 1–17 individuals accounted for the majority, with 58.3% (331,758) present in a single individual and progressively fewer shared across larger subsets. In contrast, NC samples displayed fewer highly shared clonotypes, with most sequences confined to single or few individuals. After excluding sequences also detected in NC, we identified 247 clonotypes shared by all 17 Myo patients, and 12,606 clonotypes shared by at least11 patients (**Figure 4C**), suggesting potential disease-associated public TCRs.

### Annotation of TCR-β CDR3 Sequences

**Figure 5:**
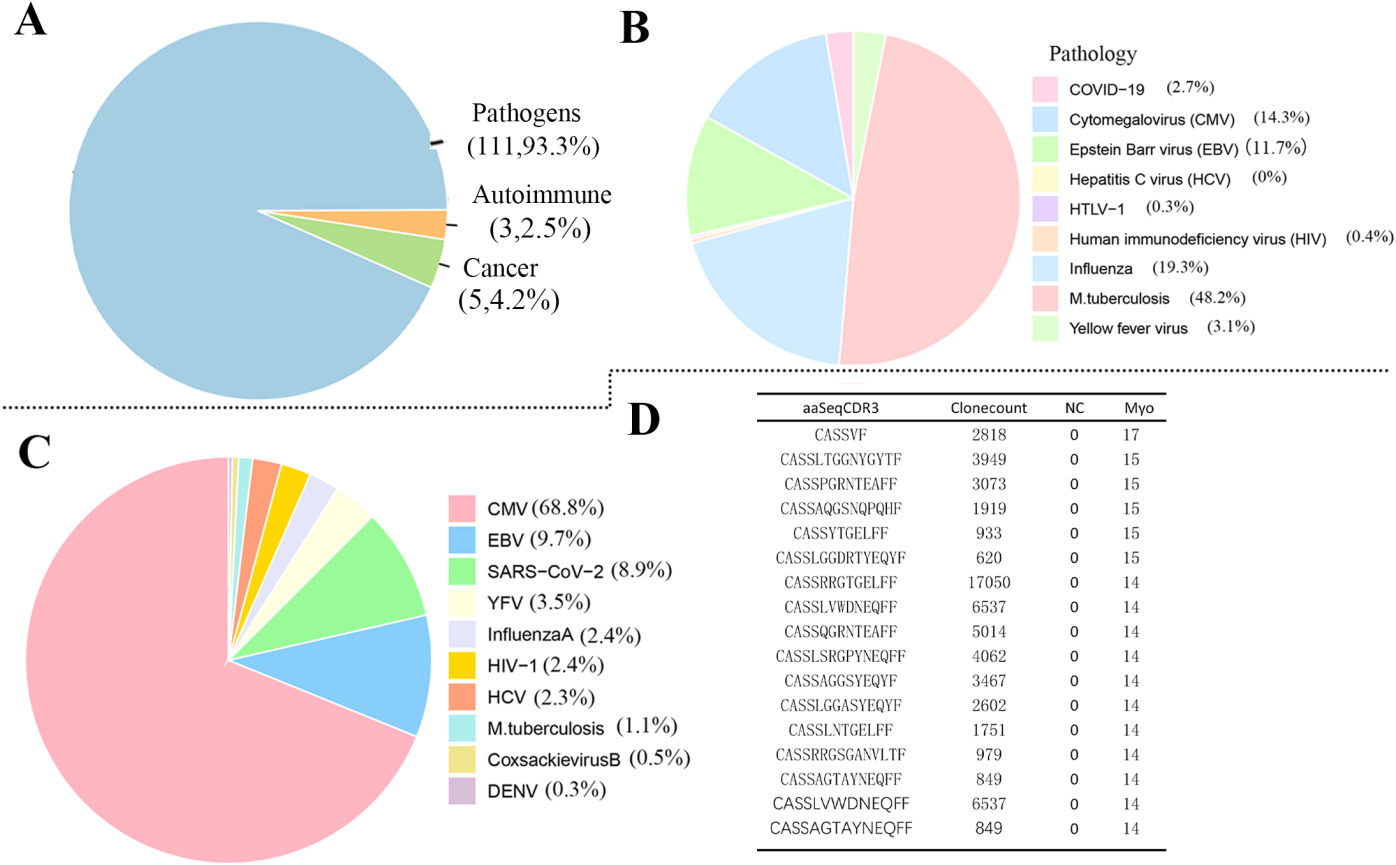
Annotation of shared clonotypes in the Myo group. **(A)** McPAS-TCR annotation of clonotypes shared by ≥11 Myo samples but absent from NC controls, showing associations with pathogens, autoimmunity, and cancer; **(B)** Distribution of pathogen-associated clonotypes from **(A)**, highlighting Mycobacterium tuberculosis, influenza virus, cytomegalovirus, and Epstein–Barr virus; **(C)** VDJdb annotation revealing associations with multiple viral pathogens, including cytomegalovirus, EBV, SARS-CoV-2, yellow fever virus, hepatitis C virus, HIV-1, and dengue virus; **(D)** Top 17 high-frequency clonotypes extracted as candidate myocarditis-associated TCR signatures. Myo, pediatric myocarditis; NC, normal control.

To investigate potential antigenic associations, we annotated shared clonotypes present in ≥11 Myo samples but absent from NC controls using McPAS-TCR and VDJdb databases. McPAS-TCR annotation (**Figure 5A**) revealed that 93.3% of database-matched entries were reported in pathogen-related records, with smaller proportions found in autoimmunity (3.25%) and cancer (4.2%). Among pathogen-related records, entries corresponding to Mycobacterium tuberculosis was most frequent (48.2%), followed by influenza virus (19.3%), cytomegalovirus (14.3%), and Epstein–Barr virus (11.7%) (**Figure 5B**). VDJdb annotation (**Figure 5C**) retrieved database records associated with cytomegalovirus, EBV, SARS-CoV-2, yellow fever virus, hepatitis C virus, HIV-1, and dengue virus. From these analyses, we prioritized 17 recurrent clonotypes as candidate myocarditis-related TCR signatures based on their database matches (**Figure 5D**).

## Discussion

In pediatric myocarditis, we observed significant remodeling of the TCR β CDR3 repertoire, including reduced clonal diversity, expansion of large clones, skewed V/J gene usage, and enrichment of public clonotypes. These findings provide the first systematic characterization of T cell repertoire changes in pediatric myocarditis and offer insights into disease-associated adaptive immune responses. Previous studies have confirmed that the histopathological hallmark of myocarditis involves infiltration of T lymphocytes into the myocardium (23). Given that T cell antigen recognition is mediated by the TCR, particularly its variable (V) region, it is essential to consider the structural determinants of antigen specificity (24). Structurally, CDR1 and CDR2 regions are directly encoded by germline V genes, whereas the CDR3 region arises from somatic rearrangement of V, (D), and J segments, representing the major source of TCR diversity (25). In disease settings, CDR3 sequences of the TCR β chain can be activated by specific antigens, leading to rapid repertoire remodeling and adaptive immune responses (26–28). Most investigations of the TCR β CDR3 repertoire in myocarditis have been limited to adults, such as studies on immune checkpoint inhibitor–related myocarditis or autoimmune myocarditis (29, 30). To our knowledge, our study is the first to demonstrate that the TCR β CDR3 repertoire undergoes reconstruction during the onset of pediatric myocarditis. Tracking the TCR repertoire provides new insights into the relationship between T lymphocytes and pediatric myocarditis.

Pediatric myocarditis patients exhibited significantly lower Chao1 and D50 indices compared with healthy controls. Furthermore, their clonal distribution was skewed, with reduced representation of small clones and an expansion of large clones. These findings suggest a reduced diversity and a more uneven distribution of the TCR repertoire in pediatric myocarditis. Such repertoire contraction is indicative of antigen-driven clonal expansion, likely reflecting an ongoing or recent immune response against myocardial antigens or viral triggers. Previous work has shown that the TCR repertoire can undergo rapid reorganization to respond to endogenous and exogenous stimuli, thereby mediating immune surveillance and regulation (31). In the context of myocarditis, reduced repertoire diversity may limit the breadth of T cell–mediated immune surveillance while favoring the dominance of expanded effector clones, potentially contributing to immune-mediated myocardial injury. Reduced repertoire diversity has been linked to impaired peripheral immune surveillance (32). For example, loss of naïve T cell pools and repertoire diversity predicts poor vaccine responses and represents a hallmark of immunosenescence in primates (33–35).

The diversity of V and J gene usage is another key determinant of repertoire heterogeneity. In our study, clonal expansion of TRBV14, TRBV28, TRBJ1-1, TRBJ1-2, TRBJ1-5, TRBV1-6, and TRBJ2–2 was significantly increased in pediatric myocarditis, whereas expansion of TRBV9, TRBJ2-4, TRBJ2-5, and TRBJ2–7 was significantly reduced. In terms of V/J pairings, V14J2-1, V14J2-3, V14J2-5, V14J2-7, and V30J1–1 were enriched in patients, while V5J1-3, V5J2-1, V5J2-5, V5J2-6, and V11J2–3 were downregulated. Similar repertoire biases have been reported in other types of myocarditis. For example, infection with human herpesvirus 6 was associated with increased TRBV28 expression, whereas coxsackievirus infection was linked to enhanced TRBV14 usage (36). Moreover, reduced TRBV9 expansion has been observed in acute myocardial infarction patients (27). Our study is the first to demonstrate novel V and J gene usage patterns in pediatric myocarditis, suggesting that biased V-J rearrangements may drive T cell activation.

Analysis of CDR3 length distribution also revealed important repertoire features. In our cohort, CDR3 lengths ranged from 21 to 81 nucleotides with a peak at 45 nucleotides, consistent with typical distributions from mature peripheral T cell pools (37). Normally, CDR3 length histograms follow a Gaussian distribution with a three-base periodicity, and abnormal peaks may indicate clonal expansion (38). Both patients and controls showed a peak at 15 amino acids; however, average CDR3 lengths beyond 15 amino acids were significantly longer in myocarditis patients. This suggests that β chain CDR3 regions with more diverse amino acid sequences may contribute to antigen-specific responses in myocarditis. Differences in CDR3 trimming, nucleotide insertions, and amino acid usage further supported distinct repertoire remodeling in patients.

Public or shared CDR3 sequences across individuals are increasingly recognized as “public TCRs,” reflecting convergent immune responses to common antigens and serving as important signatures for disease pathogenesis and immunotherapy (30, 39, 40). Notably, we observed significantly higher TCR repertoire overlap in myocarditis patients compared to controls. Annotation of patient repertoires identified matches to database-reported TCRs associated with multiple pathogens, including Mycobacterium tuberculosis, influenza virus, cytomegalovirus, Epstein–Barr virus, SARS-CoV-2, yellow fever virus (YFV), hepatitis C virus (HCV), HIV-1, and dengue virus (DENV). Notably, some of these pathogens have been previously reported in the literature in the context of myocarditis, which may partially explain the database-matched enrichment observed here (23, 41–43).

Despite these novel findings, our study has several limitations. First, the sample size was relatively small, which may restrict the statistical power and generalizability of the results. Second, only peripheral blood samples collected at the time of hospitalization were analyzed, and paired myocardial tissue samples were unavailable, which limits the direct assessment of local T cell clonality within the inflamed myocardium. Third, bulk TCR repertoire sequencing was employed, which precludes analysis of paired α/β chains at the single-cell level and limits the resolution of antigen-specific clonotype identification. Finally, experimental validation of the pathogenic relevance of the identified clonotypes was not performed. In future studies, we plan to functionally validate the candidate T cell clonotypes identified in this study to determine their role in pediatric myocarditis. This will involve assessing their antigen specificity against predicted viral and cardiac autoantigens and evaluating key effector functions such as cytokine production and cytotoxic activity.

In conclusion, our study provides the first characterization of the TCR β CDR3 repertoire in pediatric myocarditis. We demonstrate that disease onset is accompanied by reduced repertoire diversity, skewed clonal expansion, altered V/J gene usage, and distinct CDR3 length and sequence features. These findings highlight the role of TCR CDR3 repertoire remodeling in pediatric myocarditis and suggest that repertoire profiling may provide a foundation for future studies aimed at characterizing disease mechanisms and exploring potential diagnostic or therapeutic approaches, pending further experimental validation.

## Data Availability

The datasets presented in this study can be found in online repositories. The names of the repository/repositories and accession number(s) can be found in the article/**Supplementary Material**.

## References

[1] Grosse-Wortmann L Holmes KW . Myocarditis in children: chasing a gold standard. JACC Cardiovasc Imaging. (2022) 15:1239–41. doi: 10.1016/j.jcmg.2022.04.007, PMID: 35798400

[2] Blauwet LA Cooper LT . Myocarditis. Prog Cardiovasc Dis. (2010) 52:274–88. doi: 10.1016/j.pcad.2009.11.006, PMID: 20109598 PMC5951175

[3] He W Zhou L Xu K Li H Wang JJ Chen C . Immunopathogenesis and immunomodulatory therapy for myocarditis. Sci China Life Sci. (2023) 66:2112–37. doi: 10.1007/s11427-022-2273-3, PMID: 37002488 PMC10066028

[4] Liu K Han B . Role of immune cells in the pathogenesis of myocarditis. J Leukoc Biol. (2024) 115:253–75. doi: 10.1093/jleuko/qiad143, PMID: 37949833

[5] Lasrado N Reddy J . An overview of the immune mechanisms of viral myocarditis. Rev Med Virol. (2020) 30:1–14. doi: 10.1002/rmv.2131, PMID: 32720461

[6] Ammirati E Moslehi JJ . Diagnosis and treatment of acute myocarditis: A review. JAMA. (2023) 329:1098–113. doi: 10.1001/jama.2023.3371, PMID: 37014337

[7] Ammirati E Veronese G Bottiroli M Wang DW Cipriani M Garascia A . Update on acute myocarditis. Trends Cardiovasc Med. (2021) 31:370–9. doi: 10.1016/j.tcm.2020.05.008, PMID: 32497572 PMC7263216

[8] Zhang L Liu K Duan X Zhou S Jia H You Y . Cxcl12/Cxcr4 axis mediates Cd8 (+) T cell overactivation in the progression of viral myocarditis. J Transl Med. (2025) 23:399. doi: 10.1186/s12967-025-06394-6, PMID: 40186195 PMC11969836

[9] Zhu H Galdos FX Lee D Waliany S Huang YV Ryan J . Identification of pathogenic immune cell subsets associated with checkpoint inhibitor-induced myocarditis. Circulation. (2022) 146:316–35. doi: 10.1161/CIRCULATIONAHA.121.056730, PMID: 35762356 PMC9397491

[10] Minervina A Pogorelyy M Mamedov I . T-cell receptor and B-cell receptor repertoire profiling in adaptive immunity. Transpl Int. (2019) 32:1111–23. doi: 10.1111/tri.13475, PMID: 31250479

[11] Luppi P Rudert W Licata A Riboni S Betters D Cotrufo M . Expansion of specific alphabeta+ T-cell subsets in the myocardium of patients with myocarditis and idiopathic dilated cardiomyopathy associated with Coxsackievirus B infection. Hum Immunol. (2003) 64:194–210. doi: 10.1016/s0198-8859(02)00798-x, PMID: 12559622

[12] Gonnella PA Waldner H Del Nido PJ McGowan FX . Inhibition of experimental autoimmune myocarditis: peripheral deletion of Tcr Vbeta 8.1, 8.2+ Cd4+ T cells in Tlr-4 deficient mice. J Autoimmun. (2008) 31:180–7. doi: 10.1016/j.jaut.2008.06.002, PMID: 18715752

[13] Liu K Zhang L Duan X Jia H Zhou S Ma M . Peripheral immune imbalance in pediatric fulminant myocarditis revealed by single-cell sequencing and plasma proteomics. Genes Immun. (2025) 26:394–412. doi: 10.1038/s41435-025-00343-5, PMID: 40629105

[14] Sur M Rasquinha MT Mone K Massilamany C Lasrado N Gurumurthy C . Investigation into cardiac myhc-alpha 334-352-specific Tcr transgenic mice reveals a role for cytotoxic Cd4 T cells in the development of cardiac autoimmunity. Cells. (2024) 13(3):234. doi: 10.3390/cells13030234, PMID: 38334626 PMC10854502

[15] Moratto D Giacomelli M Chiarini M Savare L Saccani B Motta M . Immune response in children with Covid-19 is characterized by lower levels of T-cell activation than infected adults. Eur J Immunol. (2020) 50:1412–4. doi: 10.1002/eji.202048724, PMID: 32592406 PMC7361574

[16] Jun L Lanwei Z Jiayi W Xinsheng Y . A new immunological index for the elderly: high proportion of multiple Tcr T cells based on Scrna-Seq. Aging Dis. (2024) 15:948–50. doi: 10.14336/AD.2023.0509-1, PMID: 38722790 PMC11081166

[17] Lin H Peng Y Chen X Liang Y Tian G Yang J . T cell receptor repertoire sequencing. Methods Mol Biol. (2020) 2204:3–12. doi: 10.1007/978-1-0716-0904-0_1, PMID: 32710310

[18] Bolotin DA Poslavsky S Mitrophanov I Shugay M Mamedov IZ Putintseva EV . Mixcr: software for comprehensive adaptive immunity profiling. Nat Methods. (2015) 12:380–1. doi: 10.1038/nmeth.3364, PMID: 25924071

[19] Giudicelli V Chaume D Lefranc MP . Imgt/gene-Db: A comprehensive database for human and mouse immunoglobulin and T cell receptor genes. Nucleic Acids Res. (2005) 33:D256–61. doi: 10.1093/nar/gki010, PMID: 15608191 PMC539964

[20] Shugay M Bagaev DV Turchaninova MA Bolotin DA Britanova OV Putintseva EV . Vdjtools: unifying post-analysis of T cell receptor repertoires. PloS Comput Biol. (2015) 11:e1004503. doi: 10.1371/journal.pcbi.1004503, PMID: 26606115 PMC4659587

[21] Tickotsky N Sagiv T Prilusky J Shifrut E Friedman N . Mcpas-Tcr: A manually curated catalogue of pathology-associated T cell receptor sequences. Bioinformatics. (2017) 33:2924–9. doi: 10.1093/bioinformatics/btx286, PMID: 28481982

[22] Shugay M Bagaev DV Zvyagin IV Vroomans RM Crawford JC Dolton G . Vdjdb: A curated database of T-cell receptor sequences with known antigen specificity. Nucleic Acids Res. (2018) 46:D419–D27. doi: 10.1093/nar/gkx760, PMID: 28977646 PMC5753233

[23] Sagar S Liu PP Cooper LT Jr . Myocarditis. Lancet. (2012) 379:738–47. doi: 10.1016/S0140-6736(11)60648-X, PMID: 22185868 PMC5814111

[24] Richman SA Aggen DH Dossett ML Donermeyer DL Allen PM Greenberg PD . Structural features of T cell receptor variable regions that enhance domain stability and enable expression as single-chain valphavbeta fragments. Mol Immunol. (2009) 46:902–16. doi: 10.1016/j.molimm.2008.09.021, PMID: 18962897 PMC2666936

[25] Schatz DG Swanson PC . V(D)J recombination: mechanisms of initiation. Annu Rev Genet. (2011) 45:167–202. doi: 10.1146/annurev-genet-110410-132552, PMID: 21854230

[26] Hey S Whyte D Hoang MC Le N Natvig J Wingfield C . Analysis of cdr3 sequences from T-cell receptor beta in acute respiratory distress syndrome. Biomolecules. (2023) 13(5):825. doi: 10.3390/biom13050825, PMID: 37238695 PMC10216544

[27] Freeman JD Warren RL Webb JR Nelson BH Holt RA . Profiling the T-cell receptor beta-chain repertoire by massively parallel sequencing. Genome Res. (2009) 19:1817–24. doi: 10.1101/gr.092924.109, PMID: 19541912 PMC2765271

[28] Li D Hu L Liang Q Zhang C Shi Y Wang B . Peripheral T cell receptor beta immune repertoire is promptly reconstituted after acute myocardial infarction. J Transl Med. (2019) 17:40. doi: 10.1186/s12967-019-1788-4, PMID: 30728066 PMC6366076

[29] Blum SM Zlotoff DA Smith NP Kernin IJ Ramesh S Zubiri L . Immune responses in checkpoint myocarditis across heart, blood and tumour. Nature. (2024) 636:215–23. doi: 10.1038/s41586-024-08105-5, PMID: 39506125 PMC12952943

[30] Matsumoto Y Jee Y Sugisaki M . Successful Tcr-based immunotherapy for autoimmune myocarditis with DNA vaccines after rapid identification of pathogenic Tcr. J Immunol. (2000) 164:2248–54. doi: 10.4049/jimmunol.164.4.2248, PMID: 10657681

[31] Shao L Liu Y Mei J Li D Chen L Pan Q . High-throughput sequencing reveals the diversity of Tcr beta chain Cdr3 repertoire in patients with severe acne. Mol Immunol. (2020) 120:23–31. doi: 10.1016/j.molimm.2020.01.024, PMID: 32045771

[32] Attaf M Huseby E Sewell AK . Alphabeta T cell receptors as predictors of health and disease. Cell Mol Immunol. (2015) 12:391–9. doi: 10.1038/cmi.2014.134, PMID: 25619506 PMC4496535

[33] Mittelbrunn M Kroemer G . Hallmarks of T cell aging. Nat Immunol. (2021) 22:687–98. doi: 10.1038/s41590-021-00927-z, PMID: 33986548

[34] Granadier D Iovino L Kinsella S Dudakov JA . Dynamics of thymus function and T cell receptor repertoire breadth in health and disease. Semin Immunopathol. (2021) 43:119–34. doi: 10.1007/s00281-021-00840-5, PMID: 33608819 PMC7894242

[35] Cicin-Sain L Smyk-Pearson S Currier N Byrd L Koudelka C Robinson T . Loss of naive T cells and repertoire constriction predict poor response to vaccination in old primates. J Immunol. (2010) 184:6739–45. doi: 10.4049/jimmunol.0904193, PMID: 20483749 PMC3504654

[36] Noutsias M Rohde M Goldner K Block A Blunert K Hemaidan L . Expression of functional T-cell markers and T-cell receptor Vbeta repertoire in endomyocardial biopsies from patients presenting with acute myocarditis and dilated cardiomyopathy. Eur J Heart Fail. (2011) 13:611–8. doi: 10.1093/eurjhf/hfr014, PMID: 21422001

[37] Chen YT Hsu HC Lee YS Liu H Tan BC Chin CY . Longitudinal high-throughput sequencing of the T-cell receptor repertoire reveals dynamic change and prognostic significance of peripheral blood Tcr diversity in metastatic colorectal cancer during chemotherapy. Front Immunol. (2021) 12:743448. doi: 10.3389/fimmu.2021.743448, PMID: 35095836 PMC8789675

[38] Nishio J Suzuki M Nanki T Miyasaka N Kohsaka H . Development of Tcrb Cdr3 length repertoire of human T lymphocytes. Int Immunol. (2004) 16:423–31. doi: 10.1093/intimm/dxh046, PMID: 14978016

[39] Yan P Liu Y Zhang M Liu N Zheng Y Zhang H . Reconstitution of peripheral blood T cell receptor beta immune repertoire in immune checkpoint inhibitors associated myocarditis. Cardiooncology. (2024) 10:35. doi: 10.1186/s40959-024-00230-4, PMID: 38863010 PMC11165862

[40] Matsumoto Y . Characterization of T cell receptor (Tcr) of organ-specific autoimmune disease-inducing T cells and Tcr-based immunotherapy with DNA vaccines. J Neuroimmunol. (2000) 110:1–12. doi: 10.1016/s0165-5728(00)00346-5, PMID: 11024529

[41] Patone M Mei XW Handunnetthi L Dixon S Zaccardi F Shankar-Hari M . Risk of myocarditis after sequential doses of Covid-19 vaccine and Sars-Cov-2 infection by age and sex. Circulation. (2022) 146:743–54. doi: 10.1161/CIRCULATIONAHA.122.059970, PMID: 35993236 PMC9439633

[42] Giugni FR Aiello VD Faria CS Pour SZ Cunha MDP Giugni MV . Understanding yellow fever-associated myocardial injury: an autopsy study. EBioMedicine. (2023) 96:104810. doi: 10.1016/j.ebiom.2023.104810, PMID: 37757571 PMC10550587

[43] Miranda CH Borges Mde C Matsuno AK Vilar FC Gali LG Volpe GJ . Evaluation of cardiac involvement during dengue viral infection. Clin Infect Dis. (2013) 57:812–9. doi: 10.1093/cid/cit403, PMID: 23784923

