# Supplementary material for: TCR β CDR3 repertoire remodeling in pediatric myocarditis reveals clonal expansion and disease-associated public clonotypes
Source: Front Immunol. 2026 Feb 4;17:1711681. doi: 10.3389/fimmu.2026.1711681 (PMC12913484; doi:10.3389/fimmu.2026.1711681)
Supplement: Supplementary file 1 [file DataSheet1.docx]

**Supplementary Table S1.** Primer sequences used for human TCR β CDR3 repertoire

|  | Primer sequences (5'→3') | Primer Name |
| --- | --- | --- |
| Forward primer | ACTTCACACCTGAATGCCCTGA | TRBV1 |
|  | CAGAGAAGTCTGAAATATTCGA | TRBV2 |
|  | CCTGACTCTCCAGACAAAGTTCA | TRBV3 |
|  | CCTGAATGCCCCAACAGCTCTC | TRBV4 |
|  | TACTTCAGTGAGACACAGAGAAAC | TRBV5(1) |
|  | TTCCCTAACTATAGCTCTGAGCTG | TRBV5(3-8) |
|  | ATGGCTACAATGTCTCCAGA | TRBV6 |
|  | AGGCCTGAGGGATCCGTCTC | TRBV7 |
|  | GCACAACAGTTCCCTGACTTGCAC | TRBV9 |
|  | AAAGGAGAAGTCTCAGAT | TRBV10 |
|  | GATTCACAGTTGCCTAAGGA | TRBV11 |
|  | ATCCAGCCCTCAGAACCCAGGGA | TRBV12(125) |
|  | ATTTACTTTAACAACAACGTTCCG | TRBV12(3-4) |
|  | GATCGATTCTCAGCTCAACAG | TRBV13 |
|  | CCAACAATCGATTCTTAGC | TRBV14 |
|  | CCGAACACTTCTTTCTGCTT | TRBV15 |
|  | GGAAAGATTTTCAGCTAAGTG | TRBV16 |
|  | CCTAACGGAACGTCTTCCAC | TRBV17 |
|  | GATGAGTCAGGAATGCCAAAGGAA | TRBV18 |
|  | CAGATAGTAAATGACTTTCAG | TRBV19 |
|  | TCATCAACCATGCAAGCCTGACC | TRBV20 |
|  | CTCCAAAAACTCATCCTGTACCTT | TRBV21 |
|  | CAATGCCCCAAGAACGCACCCTGC | TRBV23 |
|  | AGTGTCTCTCGACAGGCACAGGCT | TRBV24 |
|  | TCAACAGTCTCCAGAATAAGGACG | TRBV25 |
|  | TCTTGAAATACTATAGCATC | TRBV26 |
|  | TCTCGAAAAGAGAAGAGGAAT | TRBV27 |
|  | GTCTCTAGAGAGAAGAAGGAGCGC | TRBV28 |
|  | ACATATGAGAGTGGATTTGTCATT | TRBV29 |
|  | CTCTGAGGTGCCCCAGAATCTC | TRBV30 |
| Reverse primer | TTCTGATGGCTCAAACAC | TRBC |

**Supplementary Table S2.** Summary of sequencing and clonotype assembly statistics for TCR β CDR3 repertoires.

| Sample | Total sequencing reads | Successfully aligned reads | Reads used in clonotypes, percent of total | Final clonotype count | Unique clonotype count |
| --- | --- | --- | --- | --- | --- |
| my2 | 12421697 | 9354234(75.31%) | 8214632(66.13%) | 132439 | 65543 |
| my4 | 6436680 | 3483898(54.13%) | 3039750(47.23%) | 117272 | 83672 |
| my5 | 9195733 | 3707101(40.31%) | 3113772(33.86%) | 124296 | 83400 |
| myA | 13067881 | 10094992(77.25%) | 9386114(71.83%) | 499497 | 391583 |
| myB  myC  myD  myE  myF | 6657546  8576185  8883348  6635495  5258322 | 3477583(52.24%)  4872820(56.82%)  3770491(42.44%)  3632588(54.74%)  1388187(26.4%) | 2988788(44.89%)  4216926(49.17%)  3228612(36.34%)  3244712(48.9%)  1156456(21.99%) | 41499  40779  73020  45010  13806 | 20654  20540  43841  26840  7903 |
| myG  myH  myI  myJ | 4932920  6409638  6411372  4232983 | 2064080(41.84%)  2231007(34.81%)  2534685(39.53%)  982468(23.21%) | 1755841(35.59%)  1940389(30.27%)  2166563(33.79%)  540535(12.77%) | 28800  23428  22120  5893 | 16700  12427  12472  2694 |
| myK | 8836632 | 1148065(12.99%) | 543019(6.15%) | 5371 | 2191 |
| myL  myM  myN | 3626419  7749114  7128884 | 805484(22.21%)  1298771(16.76%)  10311176(14.46%) | 668489(18.43%)  913756(11.79%)  675265(9.47%) | 2547  27928  24213 | 1071  2210  3676 |

**Supplementary Table S3.** Amino acid usage statistics in the CDR3 region for each sample.

|  | A | C | D | E | F | G | H | I | K | L | M | N | P | Q | R | S | V | W | Y | T |
| --- | --- | --- | --- | --- | --- | --- | --- | --- | --- | --- | --- | --- | --- | --- | --- | --- | --- | --- | --- | --- |
| my2 | 0.102 | 0.071 | 0.025 | 0.056 | 0.104 | 0.105 | 0.012 | 0.011 | 0.008 | 0.041 | 0.003 | 0.029 | 0.031 | 0.066 | 0.038 | 0.146 | 0.020 | 0.007 | 0.059 | 0.065 |
| my4 | 0.100 | 0.071 | 0.025 | 0.058 | 0.105 | 0.100 | 0.012 | 0.009 | 0.008 | 0.043 | 0.003 | 0.031 | 0.030 | 0.066 | 0.039 | 0.151 | 0.024 | 0.007 | 0.056 | 0.061 |
| my5 | 0.100 | 0.071 | 0.026 | 0.058 | 0.105 | 0.100 | 0.012 | 0.010 | 0.008 | 0.043 | 0.003 | 0.030 | 0.031 | 0.066 | 0.039 | 0.151 | 0.024 | 0.007 | 0.056 | 0.061 |
| myA | 0.100 | 0.070 | 0.025 | 0.059 | 0.105 | 0.100 | 0.010 | 0.010 | 0.008 | 0.042 | 0.003 | 0.031 | 0.029 | 0.066 | 0.040 | 0.149 | 0.023 | 0.007 | 0.059 | 0.063 |
| myB | 0.099 | 0.070 | 0.026 | 0.056 | 0.103 | 0.111 | 0.011 | 0.011 | 0.008 | 0.044 | 0.004 | 0.027 | 0.030 | 0.065 | 0.036 | 0.145 | 0.021 | 0.007 | 0.061 | 0.067 |
| myC | 0.100 | 0.069 | 0.026 | 0.056 | 0.102 | 0.107 | 0.012 | 0.011 | 0.008 | 0.044 | 0.003 | 0.030 | 0.031 | 0.064 | 0.038 | 0.147 | 0.021 | 0.006 | 0.058 | 0.066 |
| myD | 0.101 | 0.070 | 0.026 | 0.053 | 0.102 | 0.105 | 0.014 | 0.011 | 0.009 | 0.044 | 0.003 | 0.029 | 0.032 | 0.063 | 0.040 | 0.148 | 0.021 | 0.007 | 0.058 | 0.064 |
| myE | 0.101 | 0.070 | 0.027 | 0.059 | 0.104 | 0.102 | 0.011 | 0.010 | 0.007 | 0.041 | 0.004 | 0.030 | 0.030 | 0.066 | 0.035 | 0.149 | 0.021 | 0.007 | 0.060 | 0.066 |
| myF | 0.102 | 0.071 | 0.025 | 0.054 | 0.103 | 0.104 | 0.013 | 0.009 | 0.008 | 0.045 | 0.003 | 0.029 | 0.031 | 0.067 | 0.039 | 0.154 | 0.019 | 0.006 | 0.057 | 0.061 |
| myG | 0.100 | 0.070 | 0.026 | 0.057 | 0.104 | 0.101 | 0.011 | 0.009 | 0.007 | 0.046 | 0.003 | 0.028 | 0.029 | 0.066 | 0.036 | 0.153 | 0.020 | 0.007 | 0.061 | 0.065 |
| myH | 0.100 | 0.070 | 0.026 | 0.055 | 0.105 | 0.104 | 0.013 | 0.009 | 0.008 | 0.046 | 0.003 | 0.029 | 0.032 | 0.066 | 0.037 | 0.149 | 0.018 | 0.008 | 0.060 | 0.065 |
| myI | 0.104 | 0.068 | 0.026 | 0.055 | 0.101 | 0.100 | 0.010 | 0.011 | 0.007 | 0.046 | 0.003 | 0.029 | 0.036 | 0.066 | 0.041 | 0.146 | 0.019 | 0.006 | 0.057 | 0.070 |
| myJ | 0.104 | 0.070 | 0.026 | 0.055 | 0.103 | 0.104 | 0.014 | 0.009 | 0.007 | 0.042 | 0.004 | 0.028 | 0.029 | 0.068 | 0.036 | 0.152 | 0.018 | 0.008 | 0.057 | 0.068 |
| myK | 0.105 | 0.071 | 0.028 | 0.055 | 0.103 | 0.100 | 0.011 | 0.008 | 0.005 | 0.046 | 0.003 | 0.030 | 0.034 | 0.067 | 0.037 | 0.149 | 0.017 | 0.008 | 0.060 | 0.064 |
| myL | 0.101 | 0.069 | 0.022 | 0.052 | 0.104 | 0.097 | 0.013 | 0.008 | 0.006 | 0.049 | 0.002 | 0.030 | 0.033 | 0.067 | 0.041 | 0.165 | 0.017 | 0.005 | 0.058 | 0.060 |
| myM | 0.104 | 0.070 | 0.021 | 0.050 | 0.107 | 0.107 | 0.013 | 0.007 | 0.007 | 0.047 | 0.003 | 0.032 | 0.035 | 0.065 | 0.034 | 0.153 | 0.016 | 0.007 | 0.060 | 0.063 |
| myN | 0.097 | 0.068 | 0.026 | 0.057 | 0.103 | 0.103 | 0.015 | 0.008 | 0.008 | 0.045 | 0.002 | 0.031 | 0.034 | 0.067 | 0.040 | 0.153 | 0.019 | 0.005 | 0.054 | 0.065 |
| NC1 | 0.100 | 0.070 | 0.025 | 0.061 | 0.101 | 0.101 | 0.010 | 0.010 | 0.008 | 0.042 | 0.003 | 0.029 | 0.030 | 0.070 | 0.039 | 0.151 | 0.021 | 0.007 | 0.063 | 0.061 |
| NC2 | 0.098 | 0.070 | 0.028 | 0.059 | 0.103 | 0.093 | 0.011 | 0.011 | 0.009 | 0.044 | 0.003 | 0.029 | 0.030 | 0.069 | 0.040 | 0.150 | 0.022 | 0.007 | 0.062 | 0.063 |
| NC3 | 0.102 | 0.070 | 0.025 | 0.054 | 0.103 | 0.098 | 0.012 | 0.010 | 0.009 | 0.045 | 0.003 | 0.029 | 0.033 | 0.064 | 0.038 | 0.155 | 0.020 | 0.007 | 0.060 | 0.063 |
| NC4 | 0.100 | 0.069 | 0.024 | 0.060 | 0.102 | 0.099 | 0.009 | 0.010 | 0.007 | 0.043 | 0.002 | 0.028 | 0.032 | 0.069 | 0.039 | 0.156 | 0.020 | 0.006 | 0.064 | 0.060 |
| NC5 | 0.100 | 0.070 | 0.026 | 0.057 | 0.105 | 0.094 | 0.011 | 0.011 | 0.009 | 0.043 | 0.003 | 0.031 | 0.032 | 0.069 | 0.040 | 0.152 | 0.021 | 0.006 | 0.058 | 0.062 |
| NC6 | 0.103 | 0.070 | 0.026 | 0.060 | 0.103 | 0.100 | 0.009 | 0.009 | 0.008 | 0.047 | 0.003 | 0.026 | 0.029 | 0.067 | 0.035 | 0.160 | 0.018 | 0.006 | 0.063 | 0.059 |
| NC7 | 0.101 | 0.069 | 0.026 | 0.057 | 0.099 | 0.100 | 0.011 | 0.010 | 0.008 | 0.043 | 0.002 | 0.028 | 0.031 | 0.070 | 0.039 | 0.153 | 0.021 | 0.007 | 0.063 | 0.061 |
| NC8 | 0.101 | 0.070 | 0.028 | 0.060 | 0.102 | 0.101 | 0.011 | 0.010 | 0.008 | 0.042 | 0.003 | 0.029 | 0.031 | 0.065 | 0.038 | 0.149 | 0.022 | 0.006 | 0.060 | 0.064 |
| NC9 | 0.099 | 0.070 | 0.024 | 0.059 | 0.101 | 0.101 | 0.010 | 0.010 | 0.008 | 0.045 | 0.003 | 0.028 | 0.030 | 0.068 | 0.040 | 0.152 | 0.022 | 0.007 | 0.064 | 0.060 |

“my” followed by numbers and letters indicates pediatric myocarditis patients. Amino acids are denoted by their one-letter abbreviations.

**Supplementary Figure S1**


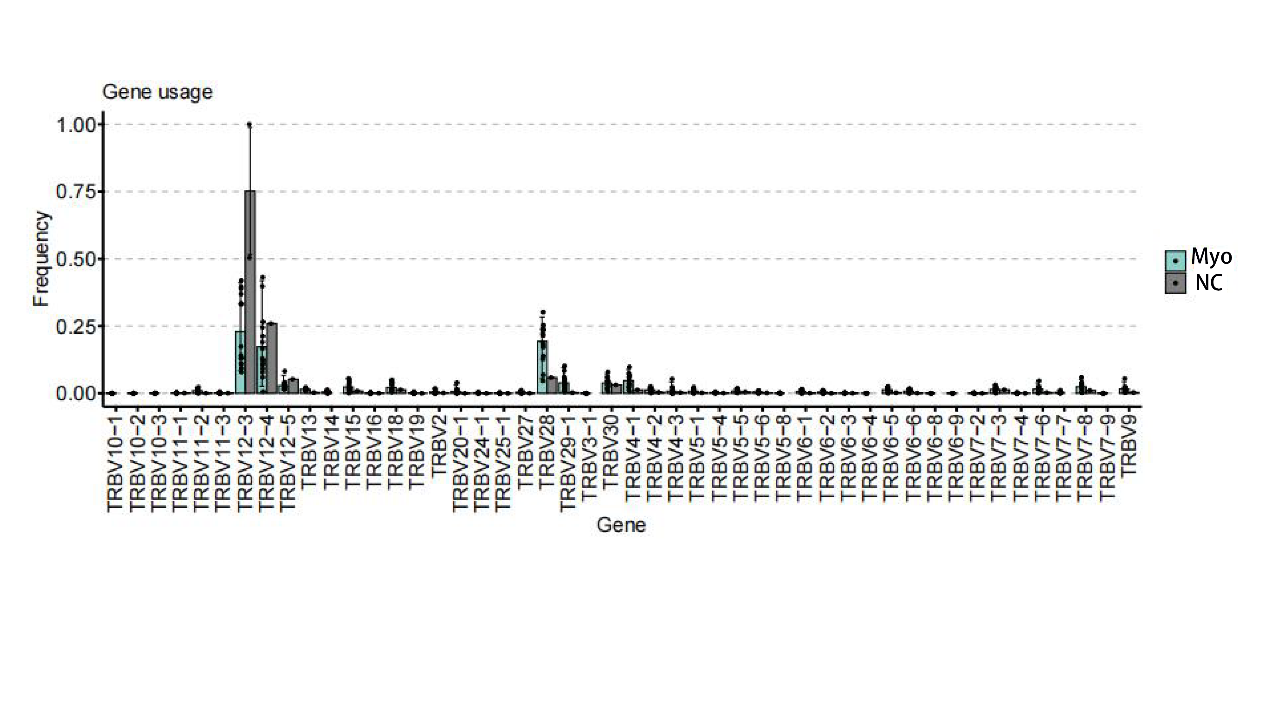


Figure S1 Statistical analysis of V gene usage

**Supplementary Figure S2**


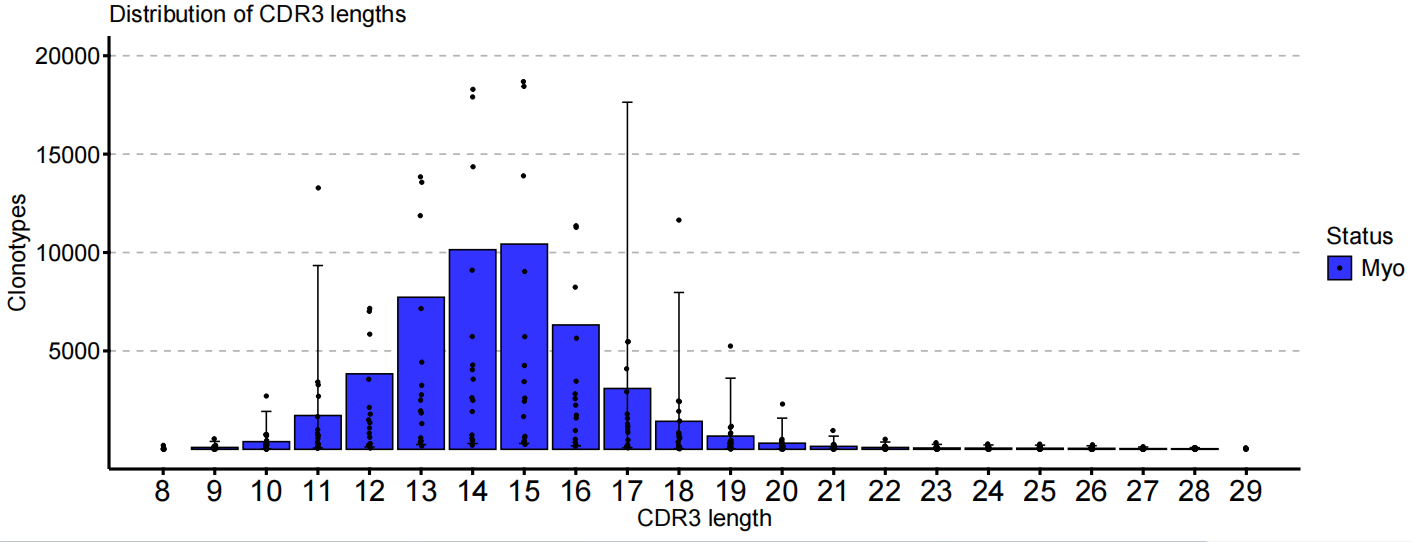


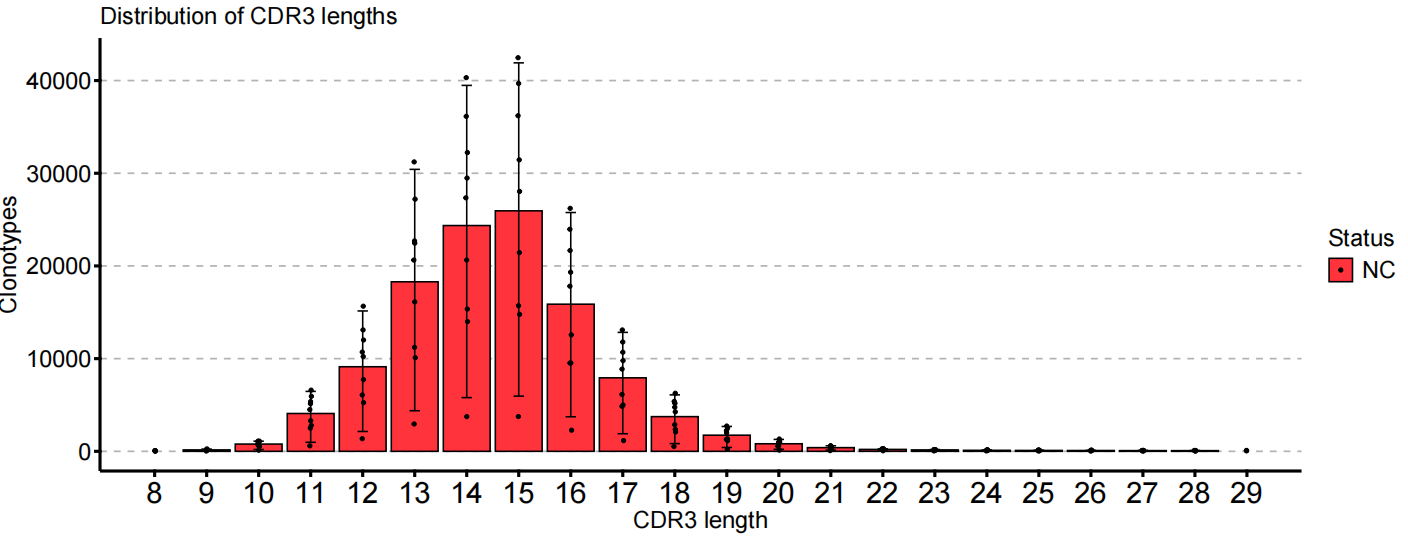


Figure S2 Statistical analysis of AA length of CDR3 region
